# Intention-based and sensory-based predictions

**DOI:** 10.1038/s41598-021-99445-z

**Published:** 2021-10-06

**Authors:** Álvaro Darriba, Yi-Fang Hsu, Sandrien Van Ommen, Florian Waszak

**Affiliations:** 1 Université de Paris, INCC UMR 8002, CNRS, F-75006 Paris, France; 2 Department of Educational Psychology and Counselling, National Taiwan Normal University, 10610 Taipei, Taiwan; 3 Institute for Research Excellence in Learning Sciences, National Taiwan Normal University, 10610 Taipei, Taiwan; 4 Department of Basic Neurosciences, University of Geneva, Biotech Campus, Geneva, Switzerland

**Keywords:** Neuroscience, Cognitive neuroscience

## Abstract

We inhabit a continuously changing world, where the ability to anticipate future states of the environment is critical for adaptation. Anticipation can be achieved by learning about the causal or temporal relationship between sensory events, as well as by learning to act on the environment to produce an intended effect. Together, sensory-based and intention-based predictions provide the flexibility needed to successfully adapt. Yet it is currently unknown whether the two sources of information are processed independently to form separate predictions, or are combined into a common prediction. To investigate this, we ran an experiment in which the final tone of two possible four-tone sequences could be predicted from the preceding tones in the sequence and/or from the participants’ intention to trigger that final tone. This tone could be congruent with both sensory-based and intention-based predictions, incongruent with both, or congruent with one while incongruent with the other. Trials where predictions were incongruent with each other yielded similar prediction error responses irrespectively of the violated prediction, indicating that both predictions were formulated and coexisted simultaneously. The violation of intention-based predictions yielded late additional error responses, suggesting that those violations underwent further differential processing which the violations of sensory-based predictions did not receive.

## Introduction

The ability to make predictions about future states of the environment allows humans to adapt their perception and optimize their behaviour. According to predictive coding models^1,2^, the brain represents predictions as probability distributions that are continuously compared with actual evidence and adjusted correspondingly. Predictions are primarily based on knowledge and experience about the relation between events in a given context. This includes causal and temporal relationships between sensory events, but also knowledge of the effects of our actions on those events. That is, we adapt to the environment by anticipating upcoming events and by producing expected events through our actions. This ability to generate sensory-based and intention-based predictions enables us to interact with our environment with great flexibility^3^. The present study aimed to use event-related potentials (ERPs) to investigate how these sensory-based and intention-based predictions interact and, particularly, whether or not they are made simultaneously and independently of each other.

Sensory-based predictions are based on global probabilities about how events happen in a given context. According to predictive coding models, the brain infers the causes of the inputs it receives and predicts future inputs accordingly^4^. Predictive processes are thought to progressively minimise the difference between predicted and actual sensory data, i.e., prediction error (PE). PE reduction is achieved through a hierarchically organized loop in which backward projections from one processing level to its subordinate provide a representation of the expected input, while reciprocal forward projections convey PE reporting the difference between that representation and the actual input^5^. Error signals are used to correct the representation, which is again provided to the subordinate level for comparison with the actual input. Sensory-based predictions are often studied employing variants of the oddball paradigm^6–10^, where deviant, oddball stimuli occur infrequently and irregularly among standard, repetitive stimuli^11^. However, since PE reduction partly manifests in these paradigms as repetition suppression, i.e., repeated stimuli generating reduced neural activity compared to novel stimuli^12–15^, designs where apprehending the probabilistic structure of the environment and anticipating an upcoming stimulus require more complex computations^16–19^ are more suitable to study sensory predictions in relation to intention-based predictions, as is the goal of the present study.

In intention-based predictions, PE is reduced by producing desired (predicted) sensory states through action (i.e., active inference^20^). This idea is closely related to ideomotor theories^21,22^, which propose that actions are represented by the sensory effects they have been experienced to produce. Performing an action would result in a bidirectional association between the action itself and its effects on the environment, which would integrate into a common code. After integration, an agent can activate this code to select the adequate motor patterns to produce a wanted effect and, conversely, anticipate the effect of a given action on the environment before executing it^23^. During action selection the predicted effect is internally anticipated or simulated. This prediction is then matched against the actual effect, the difference being sent to a higher level as PE and used to update the internal model^24,25^. Although originally formulated to explain action-unrelated sensory predictions, predictive coding models have recently been extended to the study of action-control^26^. Perhaps due to this late incorporation, research on sensory-based and intention-based predictions have followed largely separate lines.

Nevertheless, a series of brain responses have been demonstrated to occur with both types of predictions. In the present work we studied some of these responses through electroencephalography (EEG), focusing on the analyses of the N1, P2, N2b, P3a, and P3b ERP components, since they have reliably shown modulations in relation to the predictability of auditory stimuli. Sensory-based and intention-based predictions produce attenuated brain responses in the N1-P2 time range in relation to predicted stimuli (for reviews, see^3,27^). N1 is considered to reflect processing leading to conscious detection and orientation towards a sensory event^28,29^. Although the functional interpretation of P2 is debated^30^, it has been suggested to reflect higher order stimulus evaluation and classification processes^31,32^. In studies on sensory-based predictions, standard, predicted stimuli have been observed to yield attenuated brain responses in this time range compared to deviant, unpredicted stimuli, in experiments typically employing oddball paradigms^7,9,12,15,33^. Research on intention-based predictions has shown that self-generated tones elicit attenuated N1 and P2 responses in comparison to externally generated tones^34–37^. In this context, the N1b and Tb subcomponents of the N1 have been particularly related to predictions about the sensory characteristics of the stimuli, their amplitude being suppressed in response to self-generated (and therefore predicted) sounds and enhanced in response to a mismatch between the predicted and the actual auditory stimulus^36,38,39^. Interestingly, the N1–P2 attenuation does not seem to occur when actions are induced by transcranial magnetic stimulation (TMS)^40^, which suggests that it is the intention, rather than the action itself, that is necessary for generating a prediction about the effect of an action. N2b and P3 brain responses have also received substantial attention in this context. The amplitude of the N2b component, classically shown to be larger for infrequent than for frequent auditory stimuli^41,42^, has more recently been shown to be sensitive to the probability of occurrence^43,44^, as well as to conflict and mismatch detection^45,46^. Moreover, its amplitude is enhanced in response to unexpected stimuli triggered by intentional actions, thus signalling a PE response^47^. Finally, modulations in the P3 ERP responses, including P3a and P3b^48^, have been consistently observed to follow the aforementioned brain responses in both types of predictions. Amplitude enhancements of P3a have been extensively related to mismatch, surprise, and novelty processing, and consequently to PE^49–51^. P3b has also been linked to a wide variety of processes, including target detection, contextual change evaluation^52^, decision confidence^53^, and the updating of perceptual evidence^54^, all of which may arguably constitute manifestations, in different experimental setups, of a prediction updating process following PE detection. Increases of P3b amplitude have been directly associated to PE in different studies^33,55–57^.

Since the brain responses obtained from both prediction types are largely similar, one can reasonably suppose that a common, shared mechanism is responsible for their implementation. Conversely, it is also reasonable to hypothesize that these responses may depend on whether the eliciting predictions are based on sensory evidence or on the agent’s intention, since even when both predictions refer to the same event, the underlying predictive models may cover partly different aspects of it in relation to the environment. Furthermore, sensory-based and intention-based predictions often cooccur, concurring or even competing to anticipate upcoming events. It is an open question whether in these cases converging predictions integrate or, rather, are carried out in an independent manner, their effects adding up instead.

In this experiment we manipulated sensory-based and intention-based predictions independently to investigate whether or not the two sources of information generate two independent predictions in the brain. In each trial, participants were presented with one of two possible sequences of four tones, the last tone (either a high or a low tone) being predicted by the preceding ones, as taught in a series of sensory-training blocks before and between experimental blocks. Unlike previous research, we used random tone sequences as sensory-based predictive information instead of a standard oddball context to prevent repetition suppression effects. In addition, participants were cued at the beginning of each trial to generate either the high or the low tone by pressing one of two possible keys, in synchrony with the last tone of the sequence. Prior to the experimental trials, and between blocks, participants ran a series of action-training blocks in which they learned to associate the left and right keypresses with the same two high and low tones they learned as possible final tones of the sequences. Therefore, a sensory-based and an intention-based prediction converged to anticipate the last tone. Both predictions could be congruent or incongruent with each other, anticipating the same or different tones respectively. In addition, the tone eventually played could fulfil or not any of the predictions made, yielding four experimental conditions: both predictions fulfilled; both predictions violated; intention-based prediction fulfilled but sensory-based prediction violated; and intention-based prediction violated but sensory-based prediction fulfilled. Please, note that the critical comparisons involved predicted and mispredicted (rather than unpredicted) stimuli, an important differentiation given that mispredicted and unpredicted stimuli may have dissociable neurophysiological mechanisms^19,58^. We hypothesized three possible scenarios. First, one prediction prevails over the other in a winner takes it all manner. In this scenario, the pattern observed when the prevailing prediction is violated would be similar to that obtained when both predictions are violated, and the pattern observed when the prevailing prediction is fulfilled would be similar to that obtained when both predictions are fulfilled. We would then observe only one main effect. Second, both predictions are made and interact in one of two ways. In the conservative way, both predictions need to be fulfilled to avoid a PE. Therefore, error responses would be identical when both predictions are violated and when sensory-based and intention-based predictions compete (one fulfilled, one violated), compared to when both predictions are fulfilled. In the liberal way, only one prediction needs to be fulfilled to avoid a PE. Consequently, error responses would be the largest when both predictions are violated, and identical and smallest when sensory-based and intention-based predictions compete and when both predictions are fulfilled. In these two cases we would observe the corresponding interactions. Third, both sensory-based and intention-based predictions are made independently of each other and their effect on PE is additive. In this scenario, error responses would be largest when both predictions are violated and intermediate when sensory-based and intention-based predictions compete, all compared to when both predictions are fulfilled. In this last scenario we would find two independent main effects.

## Materials and methods

### Participants

A total of 21 healthy volunteers (age: mean = 21.71, SD = 1.42; 8 males; 19 right-handed) participated in the experiment with no history of neurological, neuropsychiatric, or visual/hearing impairments as indicated by self-report. All participants gave written informed consent and were paid for participation. The study was conducted in accordance with the Declaration of Helsinki and approved by the Research Ethics Committee at National Taiwan Normal University.

### Stimuli and procedures

Six sinusoidal tones were generated using Sound Forge Pro 10.0 (Sony Creative Software Inc.). The duration of each tone was 50 ms (including 5 ms rise/fall times). The frequency of each tone was within the range of 261.63–987.73 Hz, matching the absolute frequency of a series of 6 natural keys on a modern piano (i.e., C4 D5 E5 F5 G5 B5) (Table 1).

**Table 1:** Frequency of each tone.

	C4	D5	E5	F5	G5	B5
Frequency (Hz)	261.63	587.33	659.26	698.46	783.99	987.77

E-prime version 2.0 (Psychology Software Tools) was used for stimulus presentation. Auditory stimulation was delivered binaurally via headphones (Sennheiser PX200-II) with an intensity of maximum 83.3 dB (56–82.7 dBA; 65–83.3 dBC). Visual stimuli (cue and feedback) were presented at the centre of a 16-inch cathode-ray tube (CRT) screen placed at a viewing distance of 120 cm. Participants were instructed to place their thumbs on the “1” and “3” keys on a numeric keypad.

For each participant, two sequences of four tones were generated at the beginning of the experiment, according to the following restrictions: first, one of the sequences ended with the lowest tone (C4) while the other ended with the highest tone (B5); second, for each participant the preceding three tones in each sequence were randomly chosen among the other four possible tones (D5, E5, F5, G5), with the only restriction that no tones could be repeated within a sequence.

**Figure 1:**
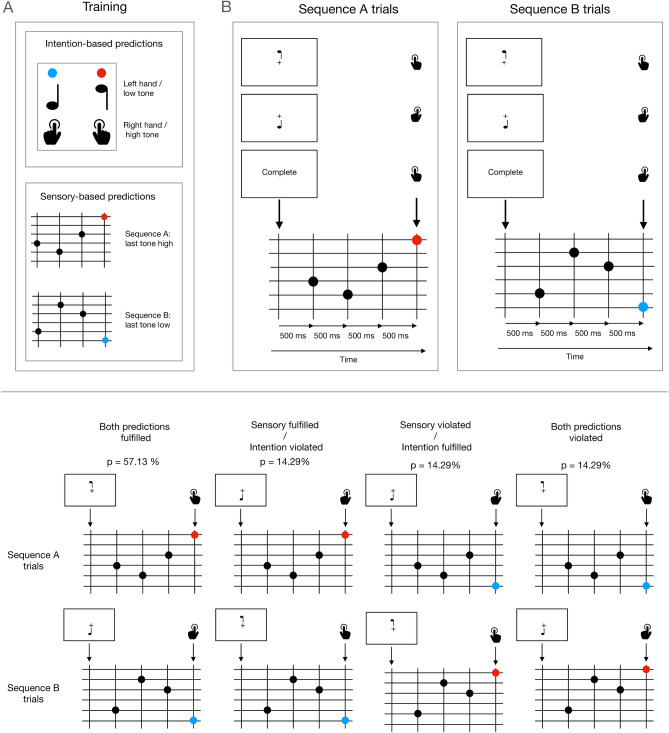
*Upper panel* Schematic description of the task. (**A**) Prior to the experiment, participants performed a series of training blocks aimed at allowing them to associate left and right keypresses with high and low tones (intention-based predictions) and to learn the two possible sequences they could be presented with (sequence A, sequence B), each ending either with the same high or low tones (sensory-based predictions) participants associated with each of the keypresses. Participants performed four blocks of 20 trials for each type of prediction. Additional, shorter training (one block of 10 trials for each type of prediction) was provided before each experimental block. (**B**) On each experimental trial, participants were presented with either sequence A or B, randomly intermingled between trials. Every trial commenced with a cue signal (500 ms), prompting participants to press a key in order to produce the last tone of the sequence in synchrony with the moment in which it should be played. This cue indicated participants to press either the key that produces the high tone, the key that produces the low tone, or the key that produces the tone that completes the sequence (catch trials). Therefore, the last tone could be predicted on the basis of the preceding sequence of tones (sensory-based predictions) and on the basis of the executed action (intention-based predictions), yielding four experimental conditions: sensory and intention predictions fulfilled (57.13% of trials), neither sensory nor intention predictions fulfilled (14.29%), sensory fulfilled but intention prediction violated (14.29%), and sensory violated but intention prediction fulfilled (14.29%). *Lower panel* Representation of the four possible experimental conditions for trials from two given sequences A and B, as indicated in the upper panel, together with the relative probabilities of each condition over the total number of trials.

Prior to the experiment, participants were presented with four sensory-based and four intention-based prediction training blocks, containing 20 trials each (Fig. 1, upper panel A). Sensory-based and intention-based blocks were presented in an alternate manner. Sensory-based blocks intended to allow participants to learn the tones that complete each of the two possible sequences they would be presented with during the experiment. Participants just looked at the screen and passively listen to each of the two possible sequences, randomly interspersed. In intention-based blocks participants were required to press one of the keys every time it was indicated on the screen, so that the keypress would trigger a given tone. These blocks aimed at allowing participants to learn the association between each one of two actions (right- or left-hand keypresses) and each one of two possible tones (the low or the high tone, i.e., C4 or B5), and thus the probability of each keypress triggering always the same tone was 100%. The hand/tone association was counterbalanced between participants. Besides the initial training blocks, participants were presented with 10 sensory-based and 10 intention-based training trials prior to every experimental block.

Figure 1 (upper panel B) shows a schematic illustration of the design. In each trial participants were presented with one of the two sequences of four tones and were required to generate the fourth and last tone by pressing one of the two keys after hearing the first three tones of the sequence. A cue presented at the beginning of each trial indicated participants whether they had to generate the high or the low tone, according to what they had learned in the action-based training blocks, independently of the presented sequence. The cue was presented for 500 ms, and consisted of one or two possible tone symbols indicating a high or a low tone. Participants were instructed about the meaning of these symbols before the experimental blocks. The tone that participants were asked to generate could be coherent or not with the tone expected to complete the sequence on the basis of what participants had learned in the sensory-based prediction training blocks. Therefore, the last tone could be coherent with the intention-based prediction, with the sensory-based prediction, with both or with none accordingly to the probabilities shown in the lower panel of Fig. 1, yielding the four possible experimental conditions. Participants were instructed to press the key to complete the sequence with approximately the same timing the tone would be played if a keypress was not required (500 ms after the previous tone). When participants pressed too quickly (less than 300 ms before the expected timing, that is, less than 200 ms after the preceding tone) or too slowly (more than 1000 ms after the preceding tone), a feedback message (“Too fast” or “Too slow”, respectively, was displayed). The next trial started 700 ms after either the tone is played or the feedback is presented. The experiment consisted of 8 blocks containing 70 trials each, for a total of 560 trials, including 320 trials with both predictions fulfilled, and 80 for each of the three other conditions (sensory-based prediction fulfilled and intention-based violated; sensory-based violated and intention-based fulfilled; both predictions violated). 80 additional catch trials were randomly intermingled with the 560 experimental trials in order to keep participants actively involved in the experiment. These trials were similar to the experimental ones with the only difference that the initial cue prompted participants to produce the tone that completed the presented sequence instead of a particularly cued one.

### EEG recording and pre-processing

EEG was recorded from 62 sintered Ag/AgCl electrodes, placed according to the extended 10–20 system on a Neuroscan quik-cap. The reference electrode was placed between Cz and CPz, and the ground electrode was set at the AFz site. Four additional electrodes were placed above and below the left eye and at the outer canthi of both eyes, and bipolarized online to register vertical and horizontal electrooculogram (EOG), respectively. A Neuroscan Synamps 2 amplifier (Compumedics Neuroscan, USA) was used to amplify and online filter (0.1–100 Hz) all signals, which were recorded at a 500 Hz sampling rate.

EEG data were processed using EEGLAB^59^ v2021.0 running under MATLAB R2020a (Mathworks, Navick, MA). Images depicting the ERP waveforms and the topographic distribution of voltage were obtained using the same software. Pre-processing was performed as follows. EEG data were re-referenced offline to linked mastoids. Data were filtered using a 0.1-Hz high-pass and 45-Hz low-pass windowed sinc finite impulse response filter (hamming window, filter order 8250 [high pass] and 166 [low pass])^60^. Bad channels were then identified by visual inspection and excluded from processing. On average, 0.52 ± 0.73 channels were removed. Epochs for each stimulus type were extracted from − 200 to + 1000 ms with respect to the target stimulus in each sequence, and were inspected for non-stereotyped artifacts and removed if present (1.70% ± 1.87 of trials removed). Stereotyped artifacts, including blinks, eye movements, and muscle artifacts were deleted via independent component analysis (ICA) using the extended infomax algorithm (Bell and Sejnowski 1995). Components containing those artifacts were rejected by visual inspection and based on measures computed with FASTER^61^, ADJUST^62^, and SASICA^63^. The average number of independent components removed was 3.45 (± 0.95 SD). The remaining components were then projected back into electrode space. On average, the minimum number of trials available for analysis was 314.6 (± 4.64 SD) in the both predictions fulfilled condition, and 78.64 (± 1.49 SD) in all the other conditions. EEG data were then transformed using a surface Laplacian filter (smoothing = 10^−5^, number of iterations = 10, spherical spline order = 4) to reduce volume conduction effects in EEG electrode space using the CSD Toolbox^64^. Finally, channels that were deemed bad were reintroduced by interpolating data between neighbouring electrodes using spherical spline interpolation^65^.

### EEG analyses

ERP analyses were performed on ICA-corrected CSD-transformed epochs time-locked to the onset of each target (− 200 to + 1000 ms). To minimize the influence of individual differences in topographies as well as the effects of performing multiple statistical comparisons, the analyses of the ERP components were performed on different ROIs of relevant sites, selected on the basis of both the grand average visual detection of the maximal peak electrodes and the topographical distribution of the activity on the scalp (see Figs. 3, 4). The time windows of interest were also determined based on the observed grand averages. Following this procedure, P2, N2b, and P3a were measured on a frontocentral cluster including FC1, FCz, and FC2 in 20 ms windows with regards to the most negative and the most positive points in the latency range of 120–180 ms, 180–220 ms, and 250–300 ms respectively. The electrodes and time windows selected for analyses are compatible with those selected in previous works^35,36,38,40,66^. Regarding N1, given that it is known to consist of several subcomponents, we ran analyses on the N1a (Na), N1b and N1c (Tb)^28,36,38,67^. Following Tonnquist-Uhlen et al.^67^, N1b was measured on C3 and C4 electrodes, where it showed its largest amplitudes (120–170 ms), and Na and Tb were identified as the first (60–110 ms) and second (120–170 ms) negative peak after stimulus onset on electrodes T7 and T8. The N1 subcomponents were analysed in 20 ms windows with regards to the most negative point in the indicated latency ranges. Finally, P3b was measured on a centroparietal cluster including CP1, CPz, and CP2 in a 50 ms window with regards to the most positive point in the latency range of 300–400 ms^68^. Baseline was designated in every case from − 200 to 0 ms relative to stimulus onset.

### Statistical analyses

Results were analysed with a Bayesian linear mixed-model (LMM) analysis using the package brms^69^, a high-level interface on Stan^70^ in R^71^. Plots were made using brms and ggplot2^72^. An advantage of LMMs over traditional approaches such as repeated measures ANOVA and paired sample t-tests is that a single model can take all sources of variance into account simultaneously. Furthermore, comparisons between conditions can easily be implemented in a single model. LMMs (of which t-tests and ANOVA are specific examples) allow for modelling complex data structures and taking correlations in data structures into account. Bayesian LMMs do so in a more powerful way than maximum likelihood models, even with small sample sizes. With weakly informative priors, Bayesian analysis gives insight in the range of possible effect sizes, reduces possible overinterpretation of sampling error, and allows for direct comparison of effect sizes. It is theoretically distinct from frequentist statistics in its inferences. The coefficient estimates are expressed in credible intervals. Credible intervals reflect the intuitive notion of the value of a parameter falling within that interval with a given probability, 95% in this case.

We used a predefined model reflecting our experimental design^73^, and we kept this model structure the same across ERP components. Participant amplitudes were normally distributed and did not need transformation to their logarithmic function^74^. Amplitudes were scaled for ease of interpretation and comparison. In the model, observations were predicted by Sensory (violated vs. fulfilled) and Intention (violated vs. fulfilled) in a full interaction. The model additionally included individual participant intercepts and slopes of Sensory and Intention in order to account for individual variation. Contrasts of all categorical factors were centred^75^, so the intercept of the model represents the grand mean. Planned pairwise comparisons were conducted via Bayesian hypothesis testing using the function Hypothesis in brms with Bonferroni correction. We used a generic weakly informative prior with mean 0 and 1 SD over the fixed effects and kept all other priors at default. We used 4 chains of 3000 iterations each per model, of which 1000 per chain were used for warm-up only, a maximum tree depth of 15 and a target acceptance rate (adapt delta) of 0.95. Convergence was verified through visual inspection of trace plots, and the Rhat of 1.00 for each parameter.

The model was specified as follows,

$$brm(formula: \, amplitude \, \sim \, sensory*intention + \left( {1 + sensory*intention|participant} \right).$$

## Results

### Behavioural performance

**Figure 2:**
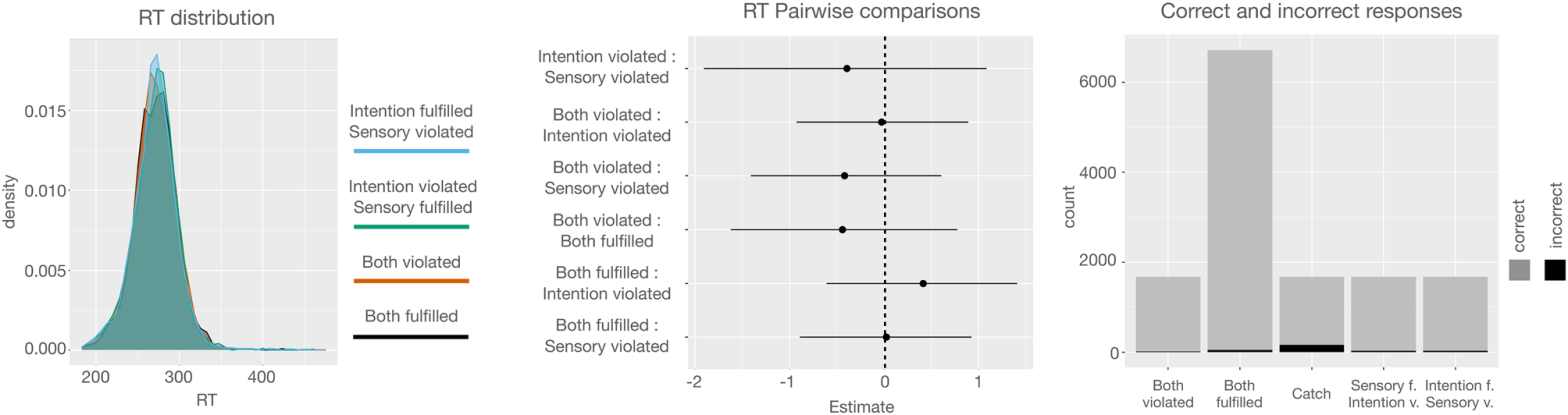
Behavioural results. Left panel shows the distribution of RTs per condition. Central panel illustrates the RT pairwise comparisons. No significant differences were observed. Right panel shows the rate of correct and incorrect responses per condition. Results indicate that participants correctly followed the instructions throughout the task. The low rate of incorrect responses in the catch trials indicates that participants were able to correctly identify the two possible two sequences.

Behavioural results (Fig. 2) showed that participants followed the instructions appropriately throughout the task, with a high rate of correct keypresses according to the initial cue and within the indicated time window (0.99 ± 0.02 SD), and similar rates across conditions: both fulfilled (0.99 ± 0.02 SD), sensory fulfilled/intention violated (0.98 ± 0.04 SD), sensory violated/intention fulfilled (0.98 ± 0.04 SD), both violated (0.99 ± 02 SD). Reaction times did not significantly differ between conditions either (both fulfilled, 272 ms ± 27 SD; sensory fulfilled/intention violated, 271 ms ± 27 SD; sensory violated/intention fulfilled, 271 ms ± 27 SD; both violated, 271 ms ± 28 SD). The rate of correct keypresses in catch trials was also high (0.9 ± 0.12 SD), showing that participants were able to learn and recognize the two possible sequences accurately.

### ERP results

**Figure 3:**
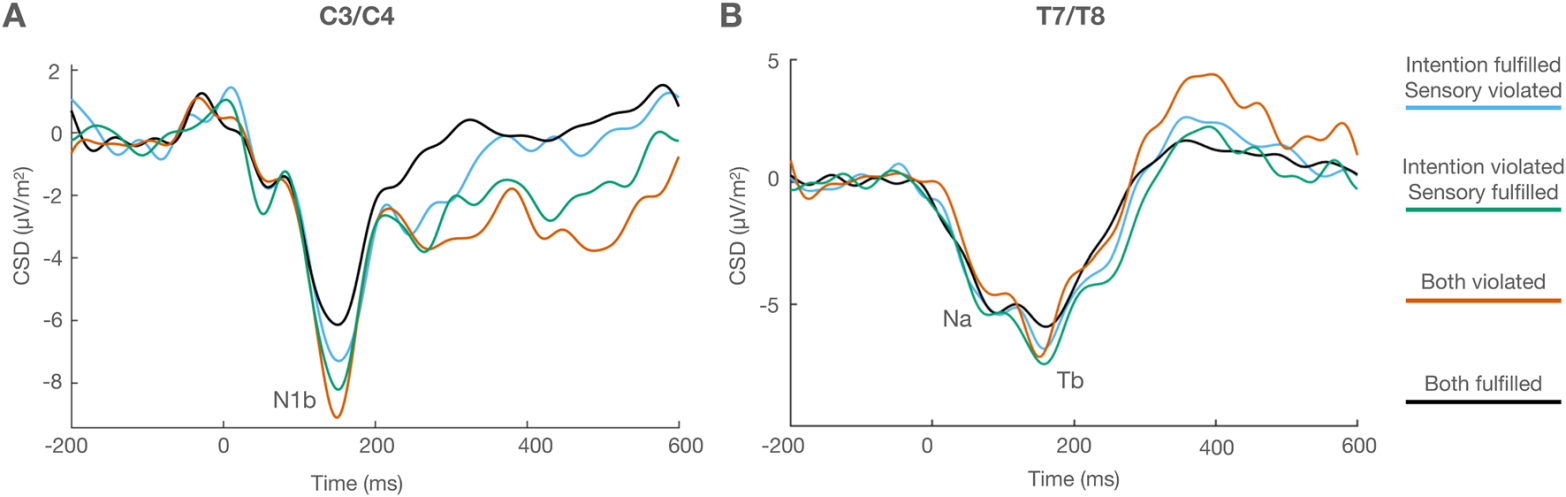
(**A**) ERP waveforms corresponding to the average of C3 and C4 electrodes, showing N1b (148 ms). (**B**) ERP waveforms corresponding to the average of T7 and T8 electrodes, depicting Na (80 ms) and Tb (158 ms).

**Figure 4:**
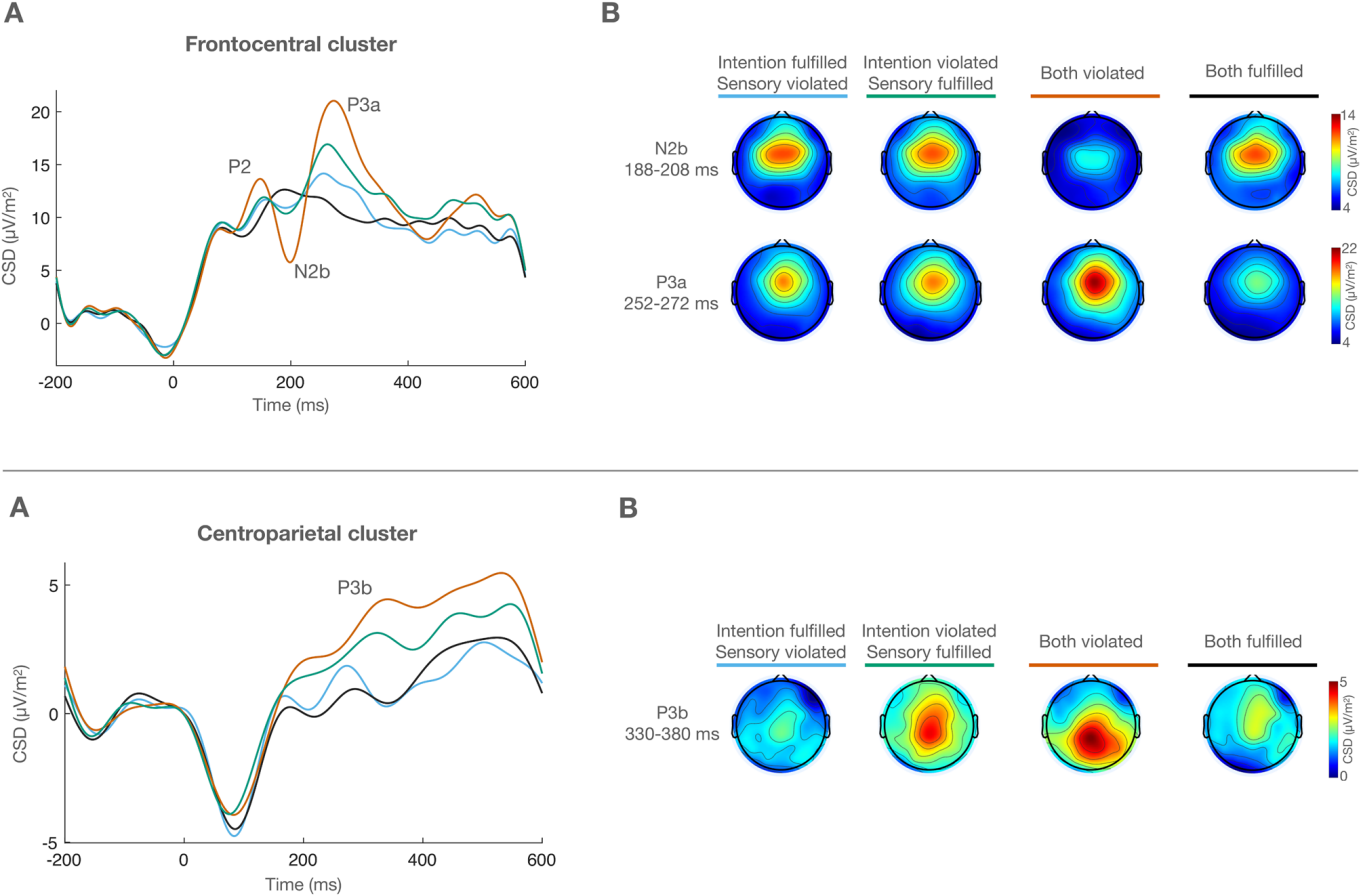
*Upper panel* (**A**) ERP waveforms corresponding to the frontocentral electrode cluster, showing P2 (between 120 and 180 ms), N2b (198 ms) and P3a (262 ms). Note that the P2 peak is delayed in the both fulfilled condition, as later confirmed by the statistical analyses. (**B**) Scalp voltage distribution maps of N2b and P3a for every condition. *Lower panel* (**A**) ERP waveforms corresponding to the centroparietal electrode cluster. P3b peaked at 356 ms. (**B**) P3b scalp voltage distribution maps for every condition.

**Figure 5:**
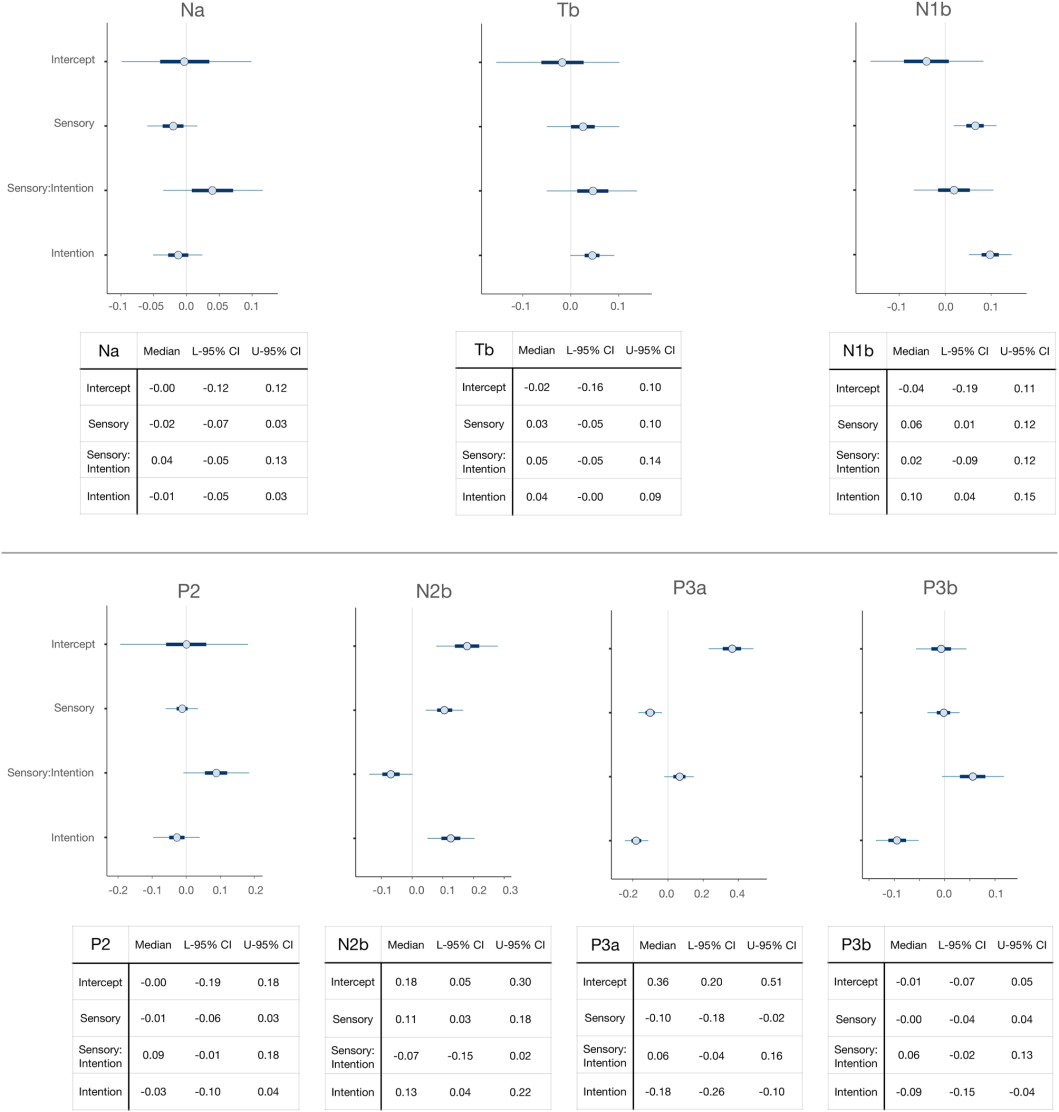
Medians and credible intervals (On the plots: 50%, thick line; 90% thin line. On the tables: 95%) of parameter values in Na, Tb, N1b, P2, N2b, P3a, and P3b. Intervals that do not include zero have the denoted probability to be a true effect.

**Figure 6:**
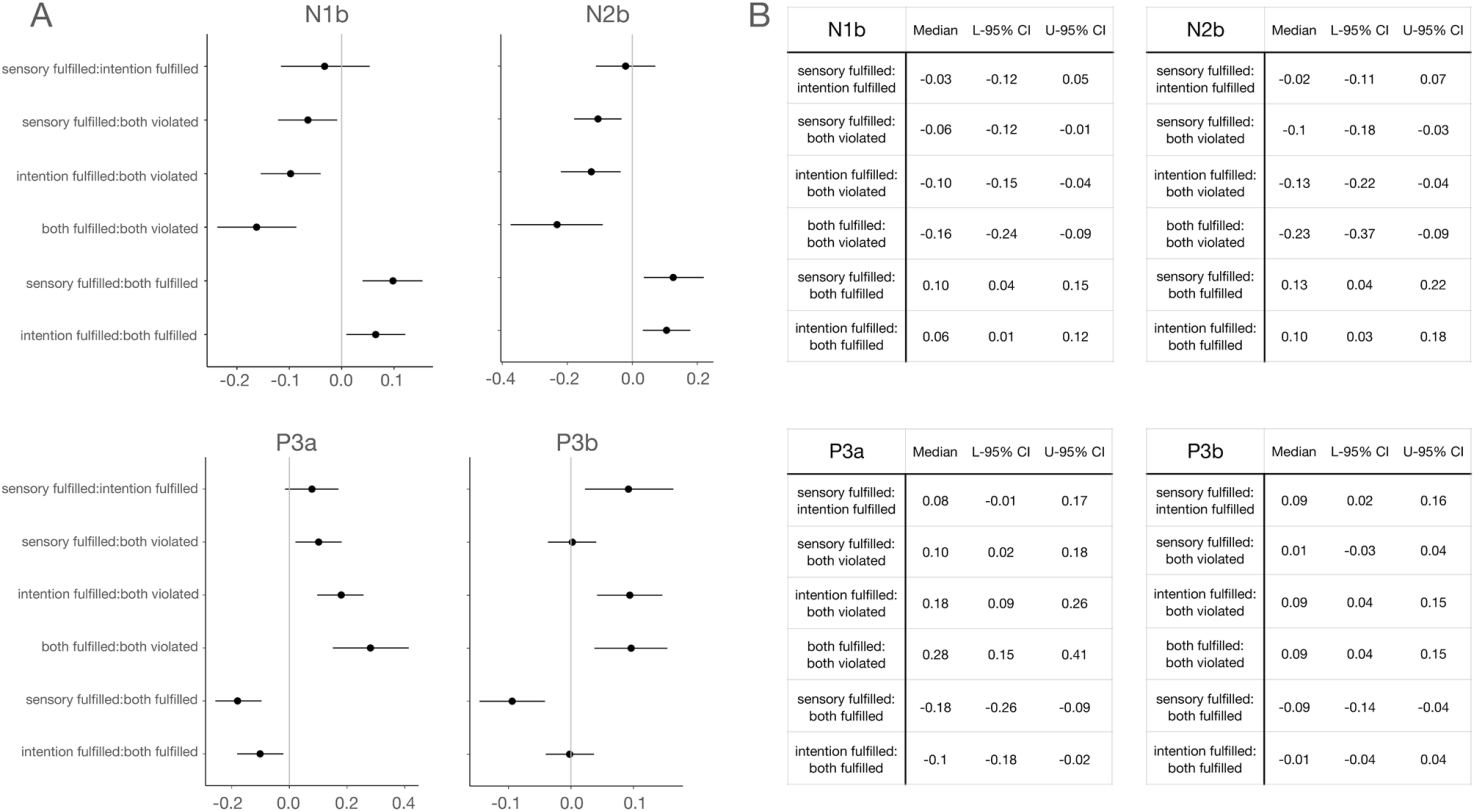
Medians and credible intervals (95%) of planned pairwise comparisons of condition estimates, calculated with the function ‘hypothesis’ in the R package ‘brms’, for components in which significant effects were observed. Intervals that do not include zero have the denoted probability to be a true effect, i.e., differences between the indicated factors to be significant. Note that the labels have been simplified for the sake of simplicity, so that sensory fulfilled refers to the sensory fulfilled/intention violated condition, and intention fulfilled to the sensory violated/intention fulfilled condition.

Figure 3 depicts the ERP waveforms at the average of C3 and C4 electrodes, where N1b reached its largest amplitude (peaking at 148 ms), and the ERPs at the average of T7 and T8, where Na (peaking at 80 ms) and Tb (peaking at 158 ms) were identified. Figure 4 (upper panel) shows the ERP waveforms corresponding to the frontocentral cluster (FC1, FCz, FC2) where P2 (140 ms in the both violated condition, 148 ms in the sensory violated/intention fulfilled and sensory fulfilled/intention violated conditions, 176 ms in the both fulfilled condition), N2b (198 ms), and P3a (262 ms) were maximal. The lower panel of Fig. 4 displays the ERPs at the centroparietal cluster (CP1, CPz, CP2), where P3b (356 ms) reached largest amplitudes. As stated in the Introduction, we hypothesized three possible scenarios. First, one prediction prevails over the other, and violating or fulfilling the prevailing prediction generates the same pattern as simultaneously violating or fulfilling both predictions. In this scenario we would observe only one main effect. Second, both predictions are made and interact in one of two ways. In the conservative way, both predictions need to be fulfilled to avoid a PE. Consequently, error responses would be identical when both predictions are violated and when sensory-based and intention-based predictions compete (one fulfilled, one violated), compared to when both predictions are fulfilled. In the liberal way, only one prediction needs to be fulfilled to avoid a PE. Therefore, error responses would be the largest when both predictions are simultaneously violated, and identical and smallest when sensory-based and intention-based predictions compete and when both predictions are fulfilled. In these two cases we would observe the corresponding interactions. Third, both sensory-based and intention-based predictions are made independently and their effect on PE is additive. In this case, error responses would be largest when both predictions are violated and intermediate when sensory-based and intention-based predictions compete, all compared to when both predictions are fulfilled. In this last scenario we would find two independent main effects. No significant results were obtained in the analyses of the amplitudes of Na, Tb, and P2. The analyses of N1b, N2b, P3a, and P3b, did show significant results. The results obtained in the statistical analyses are graphically and numerically illustrated in Figs. 5 and 6.

#### N1b results

The analysis of N1b amplitudes revealed main Sensory and Intention effects but no significant interaction, thus indicating that sensory-based and intention-based predictions independently modulated this component’s amplitude. The planned comparisons showed that both types of predictions yielded PE responses of similar size when independently violated. Additionally, each type of prediction elicited a N1b of medium size compared to the both fulfilled and both violated conditions, with sensory-based and intention-based predictions significantly differing from the former and the latter, respectively.

#### P2 results

The analysis of P2 amplitudes did not show any significant results. However, visual inspection of the ERP waveforms suggested possible differences in the latency of this component. The analysis of the latencies revealed that this was indeed the case. A linear mixed effects model of latencies in ms, including the four-level predictor of ‘Condition’ using successive differences coding, and a participant intercept revealed a difference in latencies between conditions (β =  − 26.86, t =  − 2.94 p < 0.005). A pairwise comparison corrected with the Tukey method showed that the latency of P2 was significantly longer in the ‘both fulfilled’ than in the ‘both violated’ condition, (EM = 26.86, t = 2.94, p = 0.024) with no further differences between conditions (both violated, 140 ms; both fulfilled, 176 ms; sensory fulfilled/intention violated, 148 ms; sensory violated/intention fulfilled, 148 ms).

#### N2b results

Main Sensory and Intention effects were also observed in the analysis of N2b. As for N1b, these factors did not significantly interact, indicating that sensory-based and intention-based predictions independently modulated N2b amplitude. The planned comparisons showed, on the one hand, that the independent violation of sensory-based and intention-based predictions provoked a similar PE response and, on the other, that the magnitude of these PE responses was medium in size between the simultaneous violation of both predictions and the baseline set by their simultaneous fulfilment (i.e., both fulfilled condition).

#### P3a results

Similar to N1b and N2b, the analysis of P3a revealed main Sensory and Intention effects, and no significant interaction, pointing again to sensory-based and intention-based predictions modulating the amplitude of P3a in an independent manner. As observed in the analysis of N2b, the planned comparisons indicated that the PE responses evoked by the violation of sensory-based and intention-based predictions were similar in size. These comparisons also revealed that the amplitude of P3a was in those conditions significantly larger and smaller than when both predictions were simultaneously fulfilled or violated, respectively.

#### P3b results

A main effect of Intention, together with the absence of both a main effect of Sensory and a Sensory*Intention interaction, indicated that the amplitude of P3b was modulated by the violation of intention-based but not of sensory-based predictions, independently of whether or not a sensory-based prediction was simultaneously violated. Accordingly, the planned comparisons showed that the amplitude of P3b was significantly larger in the two conditions in which an intention-based prediction was violated (both violated, intention-based violated) than in any other condition. The size of P3b did not differ between intention-based violated and both violated, confirming that the violation of sensory-based predictions had no impact on the size of P3b.

### Test for homogeneity of variances

**Figure 7:**
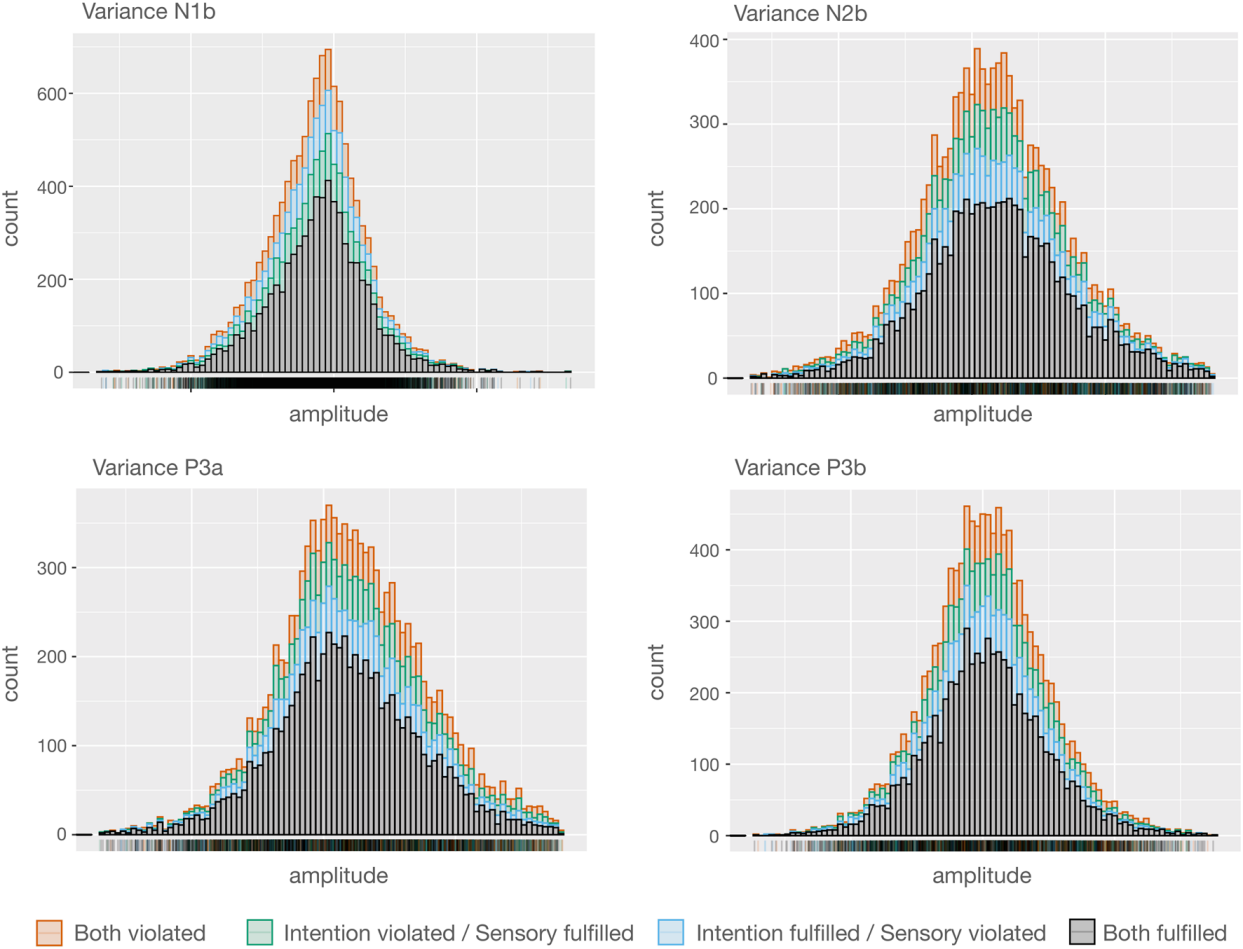
Distribution of amplitudes of each condition in stacked histograms for the four components where significant results were found. The Levene’s test confirmed that variances did not differ between conditions for each component.

The results indicate that in the conditions in which the two predictions were not congruent the violation of one of them yield error responses of medium size compared to the condition in which both predictions were fulfilled and that where both were violated (on N1b, N2b, and P3a). However, it is possible that in the conditions in which predictions are contradictory the medium error responses are due to one type of prediction prevailing over the other on a trial basis. This would cause each of these two conditions (sensory fulfilled while intention violated; sensory violated while intention fulfilled), to contain trials in which the participants’ prediction was actually violated and trials in which it was not. As a consequence, some trials would contain a PE response while some trials would not, thus yielding a reduced PE response when averaged together. In this scenario, one would expect the distribution of amplitudes across trials to differ between conditions, being more widespread or perhaps bimodal in the conditions in which only one prediction is violated compared to those in which both predictions were either fulfilled or violated. In order to rule out this possibility, we ran the Levene’s test for homogeneity of variances on the amplitudes of the four ERP components in which significant effects were found, defining variance as absolute differences from the median. These tests could not (α = 0.05) reject the null hypothesis of equal variances for N1b [F(3) = 0.78, p = 0.51], N2b [F(3) = 0.61, p = 0.61], P3a [F(3) = 2.10, p = 0.10], and P3b [F(3) = 0.61, p = 0.61], thus excluding the possibility explained above. Figure 7 depicts the amplitude distributions of each ERP component. Our results suggest that sensory-based and intention-based predictions provoked independent effects on the PE responses, and that these effects added up when both predictions were simultaneously violated.

## Discussion

In this work we investigated the interaction between sensory-based and intention-based predictions. We recorded EEG while participants performed a task in which they could predict a given stimulus on the basis of their intentional actions and/or agreeing or competing predictive sensory information. The critical comparisons thus involved predicted and mispredicted stimuli. We found two different patterns of brain activity. First, results showed main effects of sensory-based and intention-based predictions on the amplitudes of the N1b, N2b, and P3a components, and no interaction between these effects. ERP error responses were largest when both predictions concurred and were violated, compared to when both concurred and were fulfilled. When one of the two predictions was violated and the other fulfilled, results showed error responses of intermediate size. This pattern indicates that both predictions were formulated and coexisted simultaneously, so that PE was elicited when any of the predictions was violated. Second, results showed only a main effect of intention-based prediction on the amplitude of P3b. Specifically, P3b was significantly larger, compared to the trials in which both predictions were fulfilled, in those conditions in which the intention-based prediction was violated (both predictions violated, sensory-based fulfilled), but not when only the sensory-based prediction was violated. This suggests that the violation of intention-based predictions underwent further differential processing, which the violations of sensory-based predictions did not receive.

The earliest effects on the ERPs were observed as enhanced N1b amplitude when either sensory-based or intention-based predictions were violated. Interestingly, while both violations elicited a similar effect on N1b size its amplitude was significantly larger when both predictions concurrently failed. N1 has been shown to be reduced when predictions about the expected stimulus are fulfilled, and enhanced when those predictions are violated, presumably reflecting the operation of an internal predictive mechanism in sensory-based^7,8,12^ and intention-based^36,40,76^ predictions. More specifically, N1 is thought to reflect a lower-level cortical process involved in encoding simple physical features of the stimuli^8^, within a hierarchically organized deviance processing system in the auditory stream^77–79^. In agreement with those studies, the N1b modulations observed in the present experiment would reflect an early stage of PE processing, namely the detection of a discrepancy at the level of those physical features between the predicted and the actual sensory input. N1b amplitude did not differ depending on whether the violated prediction was sensory- or intention-based. This indicates that participants used both sources of information to make their predictions in a manner that rendered the final tone equally predictable on the basis of any of those sources. Whereas the effects observed on N1b largely agree with most of the literature, the lack of effects on Tb is difficult to interpret as it does not coincide with previous works in which effects on Tb were observed in response to stimuli that could be anticipated^36,38,39^. SanMiguel et al.^36^ have suggested that, in contrast to the unspecific N1 component of the auditory ERP, which seems to simply reflect orienting responses towards a sensory event, the N1b and Tb components reflect actual predictions about the sensory features of the stimuli. However, beyond this distinction, there is not a clear functional interpretation of the T-complex (in which Tb is included) in the context of prediction, and most hypotheses posed to explain N1 attenuation actually do not differentiate between N1b and Tb attenuations^30^. Even when analysed separately, they are usually discussed together. It is possible that the absence of an effect on Tb is related to the design we employed here. Most of the studies describing modulations of Tb in a predictive context have observed amplitude reduction of this component in a very specific context (of sensory attenuation), namely in response to self-generated compared to externally generated tones in a context of high regularity (where the same single tone was played in every condition throughout the experiments)^36,40^. The design employed in those studies, and the comparisons made to reveal the effects on Tb, are very different from the design we used in our experiment, where there was not a constant repetition of the same stimulus and specific predictions about what the tone would be had to be made by choosing between different alternatives, with those predictions being fulfilled or violated across trials throughout the task. Interestingly, Korka et al.^38^, employing a design aimed at investigating the interaction of sensory-based and action-based predictions, based on a high contextual regularity, found N1b enhancements in response to violations of sensory-based (regularity-based) and concurrent sensory- and action-based predictions, but, contrary to what could be expected on the basis of self-generation studies, Tb enhancements for the violations of sensory-based (regularity-based) predictions, but not for violations of action-based or simultaneous sensory- and action-based predictions. The results obtained in the studies cited above suggest that Tb modulations are particularly sensitive to contexts of high perceptual regularity. Although difficult to interpret, the absence of Tb modulations observed here could be related to the significantly lower contextual regularity in the design we employed, compared to those previous works.

P2 followed N1 in time. The literature on P2 has provided mixed results^80^, with some studies showing the enhancement or attenuation of brain responses in the P2 time range in response to unpredicted or predicted events on sensory-based^7,9,12,15,19,33^ and intention-based predictions^34–37^, and others failing to show such effects^80^. It has been proposed that P2 modulations would represent a second stage in the formation of a prediction, so that while the N1 reflects PE related to a lower-level forward prediction that prepares the auditory cortex, P2 represents a more “cognitive” PE response^80^, such as the processing of more complex characteristics of the stimulus^77^, a conscious detection of the predicted stimulus^81^, or even PE attenuation during the formation of memory traces as part of the repetition positivity phenomenon^82^. Unlike previous studies, our results did not show any effect on P2 amplitude. We can speculate about the reasons for this unexpected result. On the one hand, it might indicate that the kind of processing indexed by P2 was not necessary in the experimental context we employed. On the other hand, it could be due to P2 being overlaid by a N2b component in trials where any prediction was violated compared to those where both predictions were fulfilled^80^. This latter explanation would be supported by the effect observed on P2 latency, indicating that it peaked significantly later in trials where both predictions were fulfilled, which additionally showed a very small N2b response compared to trials containing any prediction violation, and particularly to trials where both predictions were violated, which showed a very large N2b response. The early detection of the PE, as signalled by N1b, would have activated the next stages of PE detection, improving the processing of the mispredicted stimuli, more informative than the less relevant predicted ones, which would be reflected in significant N2b responses in those trials, as explained below.

The initial detection of a PE reflected in the N1b was followed by effects on the N2b component. Specifically, as observed for N1b, N2b amplitude was significantly enhanced by the violation of any prediction compared to trials in which no violation occurred. Also, similarly to N1b, the effects of the violation of both prediction types on N2b amplitude added up instead of integrating when both predictions concurred. Several negative components occurring in the time-range of N2b, such as the mismatch negativity (MMN), the error-related negativity (ERN), or the feedback-related negativity (FRN) have been related to error signalling processes in different experimental contexts, while the possible functional relationships or equivalences between them has been the object of significant discussion (for reviews on this topic, see^43,44^). Beyond this debate, N2b amplitude is known to be sensitive to the probability of occurrence of the stimuli, showing larger amplitudes in response to deviant or unexpected stimuli^41,83^, and to require attention to the eliciting stimulus in order to be triggered^43^. N2b modulations have been consistently shown in relation to PE resulting from violated sensory-based and intention-based predictions^35,46,47,66,84^. The modulations observed here would reflect the conscious detection and registration of a mismatch between the expected and the actual sensory event, as suggested by previous research ^36,67^. As with the above-mentioned lower-level detection process signalled by N1b, this conscious detection would result from the independent monitoring of sensory-based and intention-based predictions, as suggested by the fact that N2b amplitude was enhanced whenever any of the two predictions were violated.

The error detection stages reflected in N1b and N2b likely constitute the first steps in a processing sequence^76^, followed by further effects on the frontocentral P3a and the centroparietal P3b. P3a is considered to reflect the engagement of frontal attention mechanisms for evaluating unexpected, deviant stimuli, which presumably makes those stimuli available to consciousness and behavioural control^48,85^. According to this view, the larger P3a component to mispredicted stimuli would be the consequence of the PE manifested at earlier processing stages reaching a certain threshold and thus activating an additional processing stage, which involves an involuntary capture of attention^66,86^. We found, as with N1b and N2b, larger P3a amplitudes for stimuli mispredicted on the basis of either sensory or intention information compared to trials where both predictions were fulfilled. Our results agree with previous research, showing similar P3a enhancement signalling PE in response to violated sensory-based^86–88^ and intention-based predictions^35,81,89–91^. This indicates that the violations of both types of predictions were equally salient, triggering a similar attention orienting response aimed at further processing the deviance, as both violations were unexpected and therefore very informative for a hypothetical subsequent prediction adjustment^66,92^.

Finally, the effects described above were followed by differences in the centroparietal P3b. Unlike what we observed in the frontocentral N1b, N2b, and P3a, the amplitude of P3b was significantly larger when the intention-based predictions were violated (i.e., both predictions violated, sensory-based fulfilled/intention-based violated). However, when only the sensory-based prediction was violated the amplitude of P3b did not significantly differ from trials in which both predictions were fulfilled. Among many other processes^48^, P3b has been related to uncertainty or surprise^55,83^, to decision processes^56^, and to context-updating operations^48,52,54,93^, including the updating of an internal prediction model^94^ and, specifically, the reactivation of well-established stimulus–response links^95^. Given its relationship with context updating, P3b has often been taken as a marker that stimulus processing is completed^96^. In the present study the enhancement in the posterior P3b component would reflect the operation of such a mechanism, reactivating and re-evaluating, rather than updating^97^, the link between the motor action and the stimulus when the association action-effect is violated. This step would constitute the final step of stimulus processing in those trials.

Our data indicate that predictions based on sensory and intention information were equally efficient in anticipating an upcoming stimulus, as reflected in the amplitude modulations of N1b, N2b, and P3a. The sensitiveness of these components to PE in relation to sensory-based and intention-based predictions has been shown by previous works, as explained above, and they have been suggested to be correlates of successive processing stages within a hierarchically organized deviance detection system in the auditory domain^77,78^. According to these views, different levels of information would be serially processed at different time intervals, with simple regularities being evaluated at early processing stages and more complex and integrated features being encoded in later time intervals, the predictive signals passing through hierarchically organized regions, in accordance to predictive coding notions. Our goal was to take a further step by addressing the question of whether both types of predictions can exist simultaneously. The results obtained support this hypothesis, indicating that a specific prediction was generated independently for sensory-based (bottom-up) and intention-based (top-down) predictions, and that the input was compared to both in parallel. This would be in line with previous studies suggesting that more than one regularity representation can be active at the same time, competing until one is selected^98–100^. The mechanisms processing PE in both types of predictions seem to have operated in a largely independent manner and share similar processing stages, as shown by the effects observed on the amplitudes of N1b, N2b, and P3a. Only in the final stage of stimulus processing, marked by the P3b component, stimulus processing seemed to diverge between trials in which intention predictions were violated and trials where they were not, probably because those violations tapped into the learned relationship between motor actions and their effects, reactivating the corresponding action-effect maps for re-evaluation, as explained above.

The results presented here might appear at odds with recent data from Korka et al.^38^ who compared regularity-based (i.e., sensory-based), intention-based, and joint regularity- and intention-based predictions. These authors showed the amplitudes of N1b and Tb components to be modulated by violations of tone regularity only, while violations of either regularity-based, action-based, or both, resulted in similar effects on the amplitudes of the MMN and the P3a. However, a direct comparison of the results is difficult. Unlike in the present experiment, where the two types of prediction are manipulated orthogonally, these authors compared three different types of experimental blocks which tested, respectively, regularity-based predictions, intention-based predictions, and simultaneous regularity-based and intention-based predictions. Moreover, in these latter blocks both types of predictions were always congruent with each other, so that they were always either fulfilled or violated simultaneously. Furthermore, participants were instructed to press one of the keys in 80% of the trials in order to create a tone regularity that generated both a sensory and an intention prediction, while the other key was pressed only 20% of the times. For these and other reasons related to differences in the designs employed, more research is needed to fully understand the differences in the results. These differences are particularly relevant with regard to the hypothesis on whether or not the simultaneous violation of sensory-based and intention-based predictions has additive effects on PE. Korka et al.^38^ reported no additive PE when regularity and intention were violated concurrently (i.e., the error responses were not larger compared to when only one of the predictions was violated), which led them to suggest that the two predictions integrated into a single one. In the present experiment, however, the simultaneous violation of sensory-based and intention-based predictions did elicit larger error responses than the violation of any of the predictions alone, as reflected on the amplitudes of N1b, N2b, and P3a, thus suggesting that both predictions, rather than integrate, remained independent, with the PE from the violation of each prediction adding up to produce larger PE responses. This interpretation would be in line with another study that investigated the interaction between bottom-up predictions made on the basis of auditory regularities (one of the two possible predicted tones had an overall much higher probability than the other) and top-down predictions determined by a visual cue presented at the beginning of each trial^98^. As in our study, in each trial the two sources of information could agree or disagree in their predictions. Their results showed that the violations of bottom-up and top-down predictions provoked independent PE responses, and that those responses added to each other when both predictions were concurrently violated. Although it could be argued that the top-down predictions these authors used might not be comparable to those we employed in the present experiment^101^ (i.e., visual-auditory associations versus intention-based predictions), our results are in agreement insofar as in both cases they suggest that predictions made on the basis of bottom-up and top-down information were made simultaneously and independently from each other, rather than integrating into a single prediction. Future research should investigate whether different sources of top-down and bottom-up predictions yield similar results.

Despite our results fitting in this interpretation, there is a possible alternative account of the larger effects observed when the two predictions were concurrently violated that must be taken into account. The generation of two parallel predictions and their separate comparison with the sensory input could have been favoured by the design we employed. On the one hand, participants were given the instruction to generate one or the other tone at the beginning of each trial. On the other hand, participants could quickly learn to anticipate what the last tone in the sequence should be on a sensory basis as soon as the first tones were played. It is possible, therefore, that participants detected the incongruence between both predictions very early in the trial, maybe as soon as the sequence started. Specifically, they may have learned that the probability of both predictions being congruent is more likely than the probability of predictions being incongruent (71.42% vs. 28.58%, respectively), so that the first stimuli in the sequence could generate an immediate PE response when predicting a last tone different from that indicated by the cue. This PE could be processed before the last tone is presented, which would result in reduced PE in response to the last tone. Such processing would not occur when both predictions are congruent, which would result in larger PE when they are concurrently violated, compared to when only one is violated. In order to test this, we analysed the ERPs in response to the first stimuli in the sequence (Fig. S1). We did not find significant differences between conditions in any component, which argues against the possibility of differences in PE responses prior to the presentation of the last tone explaining the pattern of results obtained. The absence of such differences, together with results indicating that both predictions were made simultaneously and coexisted independently from each other (as shown by the PE in response to any violation when predictions are contradictory and by the statistical model, revealing independent effects of both sensory and intention predictions on the ERP amplitudes), and with results showing that P3b was modulated by intention, but not by sensory predictions, suggest the independence of both predictions and support the interpretation of the larger amplitudes observed when sensory-based and intention-based were concurrently violated as reflecting the additive effects of their respective PE. However, the absence of a statistically significant Sensory-Intention interaction does not completely exclude the actual presence of such interaction. Moreover, given the differences in the relative probabilities between conditions and the well-established sensitivity of PE to probability, we cannot rule out the contribution of those probability differences to the larger effects observed when both predictions were concurrently violated. Therefore, the interpretation of larger error responses when both predictions are violated as indicating additive effects of both types of PE should be taken with caution. Future research should be conducted to specifically address this shortcoming of the present design (Fig. S1).

To conclude, our results indicate that predictions based on sensory information and on intentional action were processed in a largely independent way, eliciting separate PE responses at different processing levels. The pattern of results obtained suggests that the predictive models underlying each prediction covered partly different aspects of the sensory event beyond the strict representation of its physical features, presumably related to the different origins of the information each prediction was built upon, in one case the understanding about how events happen in the environment, and in the other some sense of agency on what the sensory event will be. Although generating and actively maintaining separate predictions on the same given event may not seem the most efficient strategy in terms of processing resources, particularly when those are redundant, it may indeed be advantageous if, as suggested by our results, the predictive models covered partially different facets of that event. We live in a complex environment in which events are often determined by multiple causes, often contradictory, and the ability to consider multiple sources of information to make predictions may allow us to better and more accurately anticipate several possible future states of the environment, providing us with the necessary flexibility to adapt our behaviour accordingly. This becomes more important when intentional actions are involved, since agents must often monitor how events happen in their surroundings for their actions to cause the desired effect at the right time. The results presented here provide evidence in this regard, showing that agents can make and maintain separate predictions on a given event based simultaneously on sensory patterns and on the expected effects of their actions.

## Supplementary Information

Supplementary Information.

## Data Availability

All the analyses described were performed using EEGLAB functions^59^, the CSD Toolbox^64^, and R^71^, which are freely available. Data presented in this paper will be made available on request subject to a formal data sharing agreement.
