# Supplementary material for: Intention-based and sensory-based predictions
Source: Sci Rep. 2021 Oct 6;11:19899. doi: 10.1038/s41598-021-99445-z (PMC8494815; doi:10.1038/s41598-021-99445-z)
Supplement: Supplementary file 1 — Supplementary Information. [file 41598_2021_99445_MOESM1_ESM.docx]

**Supplementary material**

As stated in the discussion, the design employed in the experiment presented here may have allowed participants to detect the congruence or incongruence of the two predictions very early in each trial, maybe as soon as the tone sequence started. Specifically, participants may have learned that the probability of predictions being congruent is more likely than the probability of predictions being incongruent (71,42% vs. 28.58%, respectively), so that the first stimuli in the sequence could generate an immediate PE response when predicting a last tone different from that indicated by the cue. This PE could be processed before the last tone is presented, which would result in reduced PE in response to the last tone. Such processing would not occur when both predictions are congruent, which would result in larger PE when they are concurrently violated, compared to when only one is violated. In order to test this, we analysed the ERPs in response to the first stimuli in the sequences. Such analyses were carried out in a similar way as for the main ERP analyses in response to the last stimuli, i.e., for the same components in the same electrode clusters and time windows, and with the same statistical approach (see Methods section). No significant effects were observed in any of the components, which argues against the existence in the ERPS of a PE response elicited by the early detection of the incongruence between predictions.

The ERP waveforms and the results of those analyses are presented in the figures below.


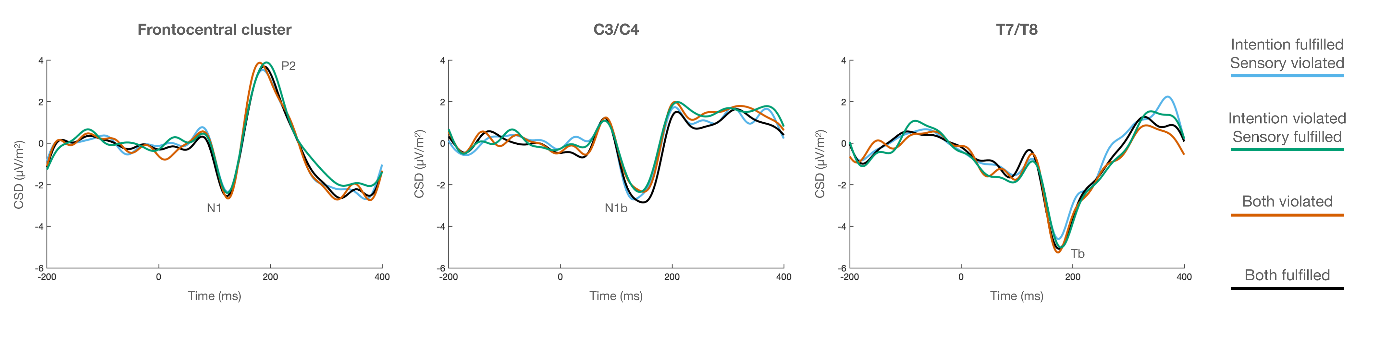


Figure 1. ERPs time-locked to the first tones in the sequences at the same electrode clusters where the main ERP analyses, time-locked to the last stimulus, were performed (see Methods section).


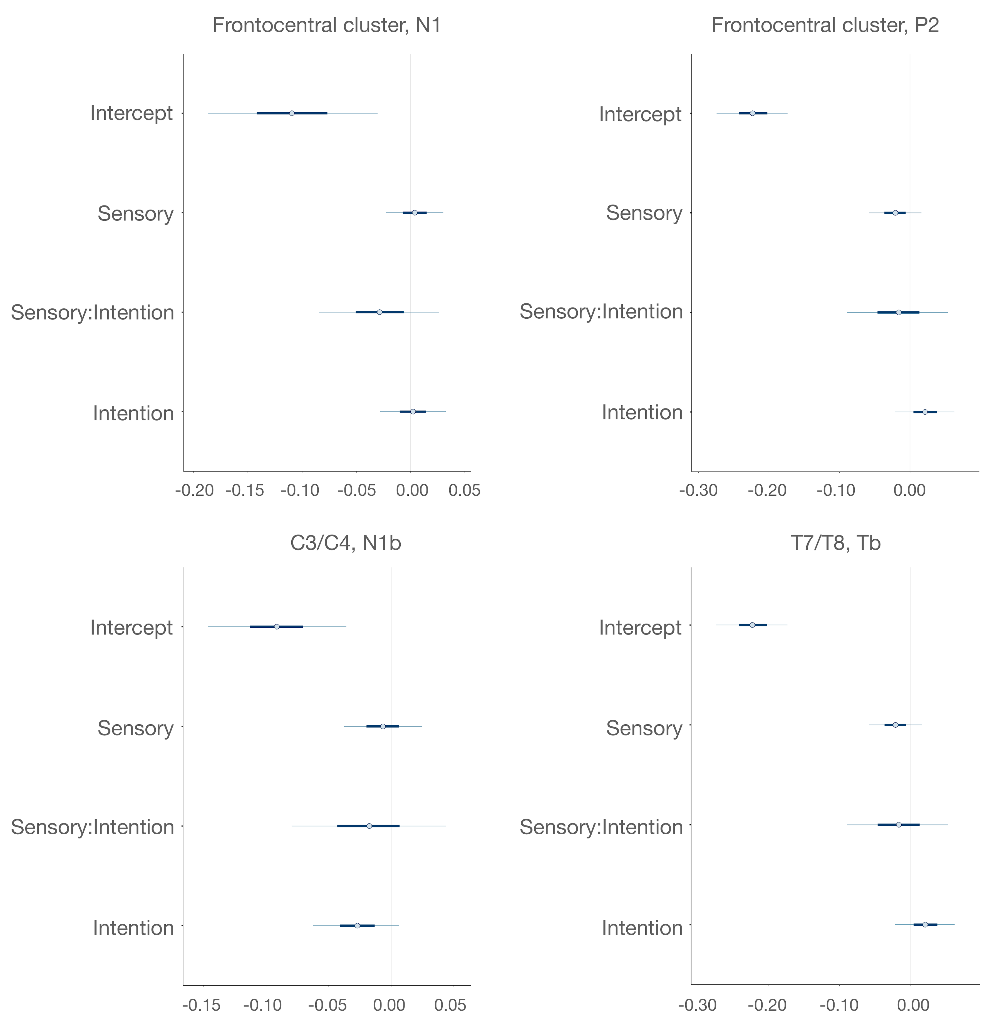


Figure 2. Statistical results obtained in the analyses of N1, P2 (Frontocentral cluster), N1b (C3, C4) and Tb (T7, T8). No significant main effects or interactions were observed.
